# Looking into the Calculating Mind: Evidence About Arithmetic from Eye-Tracking Studies

**DOI:** 10.3390/bs15121685

**Published:** 2025-12-04

**Authors:** Elisabeth Goettfried, Laura Zamarian

**Affiliations:** Department of Neurology, Medical University Innsbruck, 6020 Innsbruck, Austria; elisabeth.goettfried@student.i-med.ac.at

**Keywords:** eye-tracking, arithmetic, spatial attention, strategies, procedures

## Abstract

The investigation of eye movements has been shown to provide valuable insights into a variety of cognitive processes. A limited number of recent studies have adopted eye-tracking to investigate the processes underlying simple and complex arithmetic. Here, we review and discuss these studies. We identify two lines of research: While some studies have focused on the spatial-arithmetic associations emerging during arithmetic problem solving, some others have examined the use of procedures, strategies, and rules. Generally, results point to the added value of eye-tracking as a method for investigating different underlying processes during mental arithmetic. Altogether, eye-tracking does not only confirm the evidence gained from “pure” behavioral studies, but it also gives new insights, in particular with regard to temporal dynamics, problem difficulty, use of strategies, and inter-individual differences. Such an approach holds great potential for the studying of arithmetic not only in healthy individuals but also in clinical populations.

## 1. Introduction

Arithmetic competence is essential for effective functioning in our increasingly complex society and remains important throughout life. It extends beyond mere familiarity with numerical symbols to encompass in particular three distinct types of basic knowledge (Delazer, 2003): arithmetic fact knowledge, which enables the rapid recall of solutions to simple problems from memory (e.g., knowing that 2 × 3 equals 6); procedural knowledge, which involves the application of algorithms and strategies needed to solve more complex problems (e.g., solving 36 × 58); and conceptual knowledge, which provides deeper insight into the underlying principles and relationships within mathematics, thereby fostering flexible problem solving and reasoning. These core skills form the foundation for more advanced mathematical competencies, such as algebra and geometry. The acquisition and application of these arithmetic skills are supported by various domain-general cognitive functions, including attention, language, spatial skills, and executive control (Bull & Lee, 2014; Chow & Ekholm, 2019; Cragg et al., 2017; Hawes et al., 2022; LeFevre et al., 2010). Critically, low arithmetic competence, whether due to limited education, developmental disorders, or acquired brain damage, is associated with significant social disadvantages and an increased risk of marginalization (Butterworth et al., 2011). Consequently, gaining a deeper understanding of the cognitive underpinnings of mental arithmetic is crucial, as this provides the basis for identifying potential areas of difficulty and informing targeted, effective interventions.

Recent advances in eye-tracking methodologies offer promising avenues for exploring the complex, multi-component cognitive framework underlying arithmetic processing. Unlike traditional behavioral measures, such as reaction times (RTs), accuracy, and verbal protocols, eye-tracking can provide a real-time window into spatial attention, information processing, and problem-solving strategies as they unfold, enabling a more detailed understanding of the mechanisms involved. Eye-tracking is a versatile, non-invasive method for studying cognitive processes by recording where people look and how gaze patterns unfold during task performance (Carter & Luke, 2020; Holmqvist et al., 2011; Rayner, 2009). In the context of arithmetic, this technique can shed light on the spatial attention shifts and strategic approaches individuals employ to solve problems. This is particularly pertinent given the close links between number processing and spatial cognition (Fischer & Shaki, 2014a; Hubbard et al., 2005; Walsh, 2003; Winter et al., 2015), and between spatial attention shifts and eye movements (e.g., Altmann, 2004; Corbetta et al., 1998). It has also been demonstrated that both saccadic eye movements (a measure of overt attention orientation) and arithmetic processing activate overlapping parietal areas (Knops et al., 2009a), which provides further evidence for the connection between eye movements and number processing (e.g., Hartmann, 2022, for a review).

Modern infrared eye trackers with high spatial and temporal resolution can capture detailed gaze metrics, such as first fixation duration, fixation count, and total fixation duration (Holmqvist et al., 2011; Holmqvist & Andersson, 2017; Kasprowski, 2022), revealing how individuals engage with different aspects of arithmetic processing. While eye-tracking is well established in language and other cognitive domains (Carter & Luke, 2020; Holmqvist et al., 2011; Holmqvist & Andersson, 2017), its application to number processing and mental arithmetic is still expanding (Faulkenberry et al., 2018; Hartmann, 2022; Hartmann & Fischer, 2016; Mock et al., 2016). To date, relatively few studies have employed eye-tracking to investigate the processes involved in adult mental arithmetic in real time. This review synthetizes eye-tracking findings to argue that mental arithmetic is supported by a complex, multi-component cognitive framework. We summarize evidence from two areas—(1) spatial-arithmetic associations and (2) use of procedures, rules, and strategies during mental arithmetic—to support an integrated account of how eye movements reflect the dynamic interplay of spatial attention, mental representations, problem-solving processes, and other factors in adult arithmetic cognition. Accordingly, this review is structured as follows: First, we focus on recent eye-tracking studies exploring spatial-arithmetic associations, which capitalize on the view that eye movements are proxies for spatial attention shifts (e.g., Altmann, 2004; Corbetta et al., 1998). Then, we examine how eye-tracking informs about the use of procedures, rules, and strategies during arithmetic problem solving. For each eye-tracking study, we summarize the methodological approaches to identify possible commonalities and peculiarities that may have contributed to the results, in the view of a general discussion.

## 2. Methods

This narrative review aims to provide a targeted synthesis of two distinct, yet interconnected research areas within adult mental arithmetic: (1) spatial attention shifts, and (2) use of procedures, rules, and strategies during mental arithmetic. Rather than being exhaustive, our objective is to critically integrate existing literature to highlight key insights and explore their implications. Our search primarily relied on PubMed, employing a strategy that combined terms related to the population (“adults”) with core concepts such as (“mental arithmetic” OR “arithmetic processing” OR “calculation” OR “computation”) AND (“eye tracking” OR “gaze patterns” OR “eye movements”) AND (“spatial attention” OR “procedures” OR “strategy” OR “rules”). Additionally, we also incorporated known key authors’ names within the field and reviewed reference lists of seminal articles to identify further relevant literature. We focused on peer-reviewed journal articles published in English, primarily within the last 20 years, that provided empirical data directly relevant to our two main themes. Our search prioritized studies that reported direct eye-movement metrics—such as first fixation duration, fixation count, and total fixation duration—and excluded evidence derived from additional eye-related measures like microsaccades, pupillometry, or habituation of looking time (for an overview on these methods see Mock et al., 2016). Furthermore, we limited our scope to studies involving “healthy” adults, thereby excluding research on individuals with developmental or acquired arithmetic deficits. This focus ensures that our review pertains specifically to “normal” arithmetic processing in adulthood.

## 3. Results

### 3.1. Spatial-Numerical Associations in Arithmetic

Extensive research across diverse paradigms and populations has demonstrated robust spatial-numerical associations (for reviews, e.g., Fischer & Shaki, 2014a; Wood et al., 2008). For example, Dehaene et al. (1993) conducted a pioneering study in which participants were asked to perform a speeded parity judgement task. They found that participants responded faster to smaller numbers with their left hand and to larger numbers with their right hand. This *Spatial–Numerical Association of Response Codes* (SNARC) effect has since been replicated across various tasks, number formats, response modalities, and cultures (for reviews, Fischer & Shaki, 2014a; Wood et al., 2008). A widely accepted interpretation posits a left-to-right oriented *mental number line* (MNL), where numerical magnitudes are spatially represented with smaller numbers on the left and larger numbers on the right (for a discussion on possible origins, e.g., Fischer & Shaki, 2014a; Masson et al., 2020; Winter et al., 2015; Wood et al., 2008). The SNARC effect is just one example of the deep interconnection between numerical and spatial processing (Hubbard et al., 2005; Walsh, 2003). In this context, it has been observed that number processing can influence spatial attention: small numbers tend to direct attention to the left, while large numbers shift attention to the right (e.g., Fischer et al., 2003; for contrasting results Bonato et al., 2009). On the other hand, the presentation of visual stimuli in specific spatial locations can modulate number processing: for instance, the processing of stimuli presented in the left visual field can delay the processing of large numbers, whereas the processing of stimuli presented in the right visual field can hinder the processing of small numbers (Kramer et al., 2011; Stoianov et al., 2008). These findings support the idea that spatial-numerical associations are bi-directional. Growing evidence indicates that such associations extend beyond single-number tasks to mental arithmetic, and that the strength and expression of these associations depend on the nature and complexity of the task. Eye movements, as measured by eye-tracking and serving as overt indicators of spatial attention shifts, are particularly valuable for examining the cognitive processes involved in mental arithmetic, due to their high spatial and temporal resolution. As Fischer and Shaki (2014a) note, the role of spatial attention shifts in mental arithmetic remains unclear. Thus, eye-tracking could offer a promising means to shedding light on this question.

The following sections explore how spatial attention shifts manifest in the form of overt gaze patterns in specific mental arithmetic conditions. First, we examine the *Operational Momentum* effect, for which eye-tracking has played a pivotal role in testing whether spatial attention shifts contribute to this cognitive bias. We then consider how spatial-arithmetic associations operate on both the horizontal and vertical axes, with eye-tracking providing a direct measure of these bi-dimensional attentional shifts. Finally, we review research on the temporal dynamics of these spatial attention shifts. Here, high-resolution eye-tracking data can uniquely reveal when and under what conditions spatial attention is engaged during arithmetic problem solving.

#### 3.1.1. Operational Momentum in Mental Arithmetic

Evidence that spatial-numerical associations extend beyond single-number tasks to mental arithmetic comes from a variety of empirical findings (e.g., Fischer & Shaki, 2014a, 2014b), including the *Operational Momentum* (OM) effect. This represents a cognitive bias in approximate mental arithmetic, characterized by a tendency to overestimate addition outcomes and to underestimate subtraction outcomes. In a seminal study, McCrink et al. (2007) demonstrated the OM effect using non-symbolic arithmetic tasks. Participants viewed brief videos of object sets being added or subtracted and then judged whether the final numerosity was correct. While responses were accurate for smaller problems, larger problems revealed a clear bias: addition led to overestimation, while subtraction led to underestimation. The authors proposed that this bias arises from the directional movement implied by these operations along a logarithmically compressed MNL where participants “go too far”, resulting in underestimation or overestimation for subtraction and addition problems, respectively. Further research found similar OM effects using symbolic notations (Knops et al., 2009b; 2014), suggesting overlapping cognitive mechanisms between symbolic and non-symbolic arithmetic. An alternative account posits that the OM effect stems from attention shifts along a spatially oriented MNL (for a discussion and alternative views, e.g., Fischer & Shaki, 2014a, 2014b; Prado & Knops, 2024). According to this view, addition induces an attention “overshoot” toward larger numbers, while subtraction shifts attention toward smaller numbers (Knops et al., 2013; 2014). Under these premises, and the fact that eye fixation behavior provides more direct information about attentional shifts than deviation errors or response latencies, Klein et al. (2014) sought to evaluate the attentional shift hypothesis of the OM effect using an innovative eye-tracking paradigm involving symbolic arithmetic. In their number-to-position task, participants solved two types of problems: *operands-matched problems* (e.g., 53 + 16 = 69 and 53 − 16 = 37), where operations shared operands but yielded different results; and *results-matched problems* (e.g., 28 + 15 = 43 and 67 − 14 = 43), where different operations converged on identical results. Participants first provided verbal responses, which they then mapped onto a 0–100 number line displayed in either standard (left-to-right) or reversed (right-to-left) orientation. The study yielded two key findings. Firstly, regardless of number line orientation, a standard OM effect was observed in participants’ estimations in the operands-matched condition, whereas a reversed OM effect was observed in the results-matched condition. Secondly, deviation errors for first-fixation locations also indicated a reversed OM effect, while the directionality of further eye movements was consistent with the expected OM effect. The authors explained these findings by assuming an initial anticipation process that provided a first rough (spatial) estimate of the correct result, followed by a corrective process. As the problems from the two conditions were presented intermingled, and the anticipated location of the correct result was likely estimated to be somewhere between the correct mean results for both conditions and both operations, corrections were required in the direction associated with the respective operation for operands-matched problems (yielding a standard OM effect) and in the opposite direction for results-matched problems (producing a reversed OM effect). This study made two significant contributions. Firstly, it provided direct evidence of overt spatial attention shifts during OM effects through eye-tracking. Secondly, it provided further support to the idea that spatial-arithmetic associations are flexible and context-dependent, rather than rigidly hardwired (see also Fischer, 2012; Fischer & Brugger, 2011). These findings thus further support dynamic models of spatial-numerical associations, in which attentional processes interact with arithmetic operations in task-dependent ways.

#### 3.1.2. From Horizontal to Vertical Spatial-Arithmetic Associations

A large body of research provides further evidence for dynamic processes by reporting spatial-numerical associations across different spatial dimensions (for a review, Winter et al., 2015). Initially, these associations were established for the horizontal (left-to-right) dimension, but subsequent research has revealed similar associations along the vertical axis. In this context, smaller numbers are linked to downward space, while larger numbers are linked to upward space (Fischer & Shaki, 2014a; for a discussion on associations along the horizontal, vertical, and sagittal axes, Winter et al., 2015). These vertical associations are evident in various behavioral domains. For example, research on body motion shows that leftward and downward passive whole-body motion facilitates the random generation of small numbers, whereas rightward and upward motion facilitates the generation of large numbers (Hartmann et al., 2012). Similarly, research on eye movements indicates that a rightward and upward gaze shift tends to precede the generation of a number that is larger than the previous one, and vice versa (Loetscher et al., 2010). Not only have horizontal and vertical spatial-numerical associations been found in simple number processing, but also in mental arithmetic (for a review, Winter et al., 2015). For example, participants solved addition problems more quickly when moving upward in an elevator, and subtraction problems more quickly when moving downward (Lugli et al., 2013). Similarly, perceiving or executing downward movements interferes with addition, while upward movements interfere with subtraction (Wiemers et al., 2014).

Driven by the theoretical framework that vertical spatial-numerical associations (e.g., linking “more” with “up”) are more “grounded” in universal, embodied interactions with the physical world than the horizontal mapping (considered “situated” and shaped by cultural factors such as the reading and writing direction), Blini et al. (2019) investigated the bi-directional nature of spatial-arithmetic associations. They used optokinetic stimulation (OKS) concurrently with eye-tracking during multi-digit arithmetic tasks. OKS involves presenting full-field visual stimuli (e.g., vertical bars) that move coherently in a specific direction, which induces involuntary eye movements through optokinetic nystagmus, a compensatory reflex characterized by alternating phases of smooth pursuit and quick resetting saccades that helps stabilize the image on the retina during motion. In Blini et al.’s (2019) study, participants performed auditorily presented multi-digit addition and subtraction tasks while their eye movements were recorded through eye-tracking under different OKS conditions (left, right, up, down). Notably, the behavioral data, particularly the distribution of decade and unit errors, revealed a general pattern of underestimation for addition and overestimation for subtraction—an effect opposite to the standard OM effect (McCrink et al., 2007). Interestingly, vertical OKS, but not horizontal, modulated these effects: downward OKS improved subtraction performance by reducing overestimation errors, while upward OKS increased overestimation errors. Furthermore, the eye-tracking results showed that the arithmetic operation itself systematically influenced eye movements, even in the absence of visual “arithmetic” cues (see also Hartmann et al., 2015; Hartmann, 2022; Klein et al., 2014; Masson et al., 2018; Salvaggio et al., 2022; Zhu et al., 2018, 2019). Specifically, during horizontal OKS, subtraction (relative to addition) was associated with a leftward gaze displacement, while addition (relative to subtraction) correlated with a rightward displacement. Regardless of the OKS type, subtraction was also consistently related to a downward gaze displacement. Overall, this study not only provided further evidence of bi-directional spatial-numerical associations, aligning with prior research (e.g., Kramer et al., 2011; Stoianov et al., 2008), but also suggested that the vertical dimension has a stronger impact on mental arithmetic, as only vertical OKS systematically modulated arithmetic performance.

Against this background, Hartmann (2022) directly investigated the potential functional role of eye movements during mental calculation tasks. Here, the primary purpose of eye-tracking was to ensure compliance with the instructions. The study posed a critical question: Are the eye movements observed during calculation merely an epiphenomenon, or are they functionally part of the calculation process? To address this, Hartmann (2022) employed a rigorous paradigm that overcame key limitations of previous research by using multi-step problems and removing visual operation signs. Based on two pretests that identified exclusively vertical eye movements during addition, the main experiment required participants to verify multi-step addition problems (e.g., starting with number 59 and adding operands like 5, 4, and 3) under three eye-movement conditions: (1) fixating centrally, (2) moving their gaze freely, and (3) following a vertically moving dot either upward (congruent condition) or downward (incongruent condition). The results demonstrated that participants solved addition problems more quickly when following an upward-moving dot (congruent condition) compared to all other conditions. Crucially, the presence of a facilitation effect suggests that spatial attention shifts are not merely correlated with but functionally contribute to the computation process itself. This finding aligns with the results of Blini et al. (2019), providing compelling evidence that vertical spatial-arithmetic associations are more robust than horizontal associations, at least in complex, multi-step mental arithmetic (for discussions, Fischer & Shaki, 2014a; Winter et al., 2015). Collectively, this research emphasizes the role of spatial attention in mental arithmetic and demonstrates the unique value of eye tracking in revealing the dynamic interplay between horizontal and vertical spatial dimensions during arithmetic processing.

#### 3.1.3. Temporal Dynamics of Attention Shifts

Yet another question of interest is when and under what conditions spatial attention shifts are engaged during arithmetic problem solving. Research demonstrates that the operation sign alone can induce spatial biases. Using a speeded manual classification task, Pinhas et al. (2014) found that participants responded faster when button presses were spatially congruent with operation signs—specifically, right-sided responses for “+” and, to a lesser extent, left-sided responses for “−”—compared to incongruent pairings. This phenomenon, termed the *Operation Sign Spatial Association* (OSSA) effect, suggests that arithmetic symbols can automatically activate spatial mappings, in particular in tasks that provide contextualisation of the signs as arithmetic symbols (for a discussion, Pinhas et al., 2014). Building on these findings, Hartmann et al. (2015) sought to directly examine the temporal dynamics of spatial biases during mental arithmetic through eye-tracking and assess the nature of spatial biases in mental arithmetic. In this study, Hartmann et al. (2015) recorded spontaneous eye movements on a blank screen while participants verbally solved single-digit addition and subtraction problems (e.g., 2 + 7, 8 − 3) that were presented auditorily. Crucially, the magnitude of the first operand did not provide predictive cues about the upcoming operation, preventing anticipatory eye movements. The study uncovered three key results: (1) larger magnitudes of the operands elicited more rightward eye positions, (2) addition problems triggered more upward gaze shifts relative to subtraction before the second operand appeared—that is, prior to active computation— and (3) no horizontal gaze shifts were associated with either addition or subtraction (Hartmann et al., 2015). The authors concluded that these effects likely reflect semantic operation spatial associations rather than a mental “movement” toward the solution on the MNL. Additionally, the study suggested that, in the absence of a predefined spatial reference frame, individuals find it more intuitive to conceptualize addition and subtraction along the vertical (up-down) axis rather than along the horizontal (left-right) axis.

Extending these insights, Zhu et al. (2019) (for similar results, Zhu et al., 2018) also investigated the temporal course of spatial attention shifts in arithmetic through eye-tracking and demonstrated that these occur under specific conditions. In their study, participants solved three types of addition and subtraction problems, with elements presented successively on the screen while eye movements were recorded through eye-tracking: (1) with non-zero operands (e.g., 1 + 2, 5 − 2); (2) with the first operand zero (e.g., 0 + 2); and (3) with the second operand zero (e.g., 2 + 0, −2 + 0). Their findings corroborated prior research indicating spatial attention shifts along the horizontal axis during mental arithmetic (Knops et al., 2009a; 2014; Li et al., 2018; Liu et al., 2017; Masson et al., 2017; Mathieu et al., 2018). Specifically, they observed facilitation of rightward eye movements after addition and leftward movements after subtraction; however, these effects occurred only when both operands were non-zero or when the first operand was zero—not when the second operand was zero (for similar results: Zhu et al., 2018; for contrasting results: Masson & Pesenti, 2014; Pinhas & Fischer, 2008). Additionally, the timing between solving the arithmetic problem and detecting the visual cue significantly influenced the results, with the most pronounced effects observed at a delay of 300 msec. The authors interpreted these findings as indicating that spatial attention shifts are not merely tied to the symbols “+” or “−” but rather depend on active magnitude processing. Problems like 1 + 2 or 0 + 2 engage the MNL because they require mental quantity manipulation, whereas problems like 2 + 0 bypass magnitude processing since adding or subtracting zero does not alter the value (for contrasting results: Masson & Pesenti, 2014; Pinhas & Fischer, 2008). In summary, both studies by Zhu et al. (2018, 2019) demonstrated that the position of zero influences spatial-arithmetic associations, aligning with the view that the relationship between spatial attention and arithmetic is context-dependent.

The temporal course of attention shifts during mental arithmetic was also investigated through eye-tracking by Masson et al. (2018). Spontaneous eye movements on a blank screen were recorded while participants performed two consecutive tasks: (1) solving auditorily presented multi-digit addition or subtraction problems (e.g., 36 ± 8), followed by (2) a left-right target detection task. Half of the arithmetic problems required carrying/borrowing, while the other half did not. During the calculation period—specifically, in the time window between the presentation of the second operand and the participant’s response—their gaze shifted further rightward during addition than during subtraction. Moreover, participants were faster in detecting a rightward target after solving addition than after solving subtraction, aligning with prior findings (Liu et al., 2017; Masson & Pesenti, 2014). Regarding the source of attention shifts, Masson et al. (2018) found that the operation type (addition vs. subtraction) influenced the direction of attention shifts, whereas the magnitude of the operands or the answer did not. Additionally, no leftward deviation was observed during subtraction solving, which was explained by the use of mixed computation strategies that could have weakened—rather than strengthened—the recruitment of spatial attentional mechanisms (for a related discussion, see Masson & Pesenti, 2014). Aligning with Masson and Pesenti (2016), but contrasting with Hartmann et al. (2015), the results by Masson et al. (2018) suggest that attention shifts are functionally part of the calculation processes involved in arithmetic problem solving, as they are particularly evident in the time period after the second operand is presented (calculation period).

Similarly, Salvaggio et al. (2022) utilized eye-tracking to investigate participants as they solved auditorily presented multi-digit addition and subtraction problems. A key manipulation was the inclusion of problems that required carrying or borrowing, thereby increasing the working memory load. To facilitate the detection of number-related attention shifts, the task was paired with passive landscape viewing, serving as a concurrent, non-demanding visual task. The results revealed a rightward gaze deviation for addition relative to subtraction in two distinct time windows: the first emerged immediately after the presentation of the operator, and the second occurred during the response production phase. This effect was only significant for problems requiring carrying/borrowing, linking the attention shifts directly to the execution of specific calculation algorithms. Notably, a series of sub-analyses revealed that it was the operation, rather than the magnitude of the numbers, that was responsible for the direction of the spatial attention shifts along the horizontal axis, as gaze followed the direction implied by addition or subtraction even when number magnitudes predicted the opposite. In the vertical axis, a significant difference was observed during the response production phase, with subtraction being associated with a downward deviation relative to addition. These findings led to three key conclusions: (1) the deployment of spatial attention during mental arithmetic occurs very early (immediately after the operator is presented) and potentially contributes to narrowing down the range of possible answers; (2) the working memory load influences the involvement of spatial attention; and (3) the spatial frame used during arithmetic is bi-dimensional and can be adjusted to face the actual demands of arithmetic processing. In this framework, attention shifts are viewed as ancillary to working memory processes, actively constituting a dynamic spatial frame to meet the specific demands of arithmetic problem solving.

In summary, eye-tracking studies provide direct and converging evidence of the involvement of spatial attention shifts in mental arithmetic. The findings confirm that these shifts can occur along both the horizontal and vertical axes, are modulated by task context, operation type, and problem structure, and can manifest early in arithmetic processing. The dynamic and flexible nature of these attentional mechanisms underscores their crucial role in number cognition and supports models positing a bi-directional relationship between spatial attention and arithmetic processing, rather than a fixed, hardwired spatial-numerical mapping. Overall, eye-tracking helps to expand our understanding of the temporal and spatial dynamics of spatial attention during mental arithmetic.

### 3.2. Procedures, Rules, and Strategies in Arithmetic Problem Solving

A second line of eye-tracking studies has investigated the use of procedures, strategies, and rules during arithmetic problem solving. Arithmetic clearly involves the application of various solution methods, rules, and strategic approaches to solve problems efficiently (Kong et al., 2005; McCarthy & Warrington, 1990; van Harskamp & Cipolotti, 2001). In this context, eye-tracking emerges as a valuable tool for examining gaze patterns, enabling researchers to identify the use of specific arithmetic procedures (e.g., carrying, borrowing), rules (e.g., the *precedence* rule), and strategies (e.g., calculation vs. estimation) employed during task performance. A seminal study by Suppes et al. (1983) employed eye-tracking with five participants—comprising two adults and three children—to evaluate a proposed *register-machine* model of complex arithmetic problem solving. Their findings showed that the eye-movement data partially supported the model, revealing notable inter-individual differences in the solution steps taken (Suppes et al., 1983). Since then, various studies have leveraged eye-tracking to delve deeper into the cognitive processes underpinning arithmetic problem solving, significantly enriching our understanding of how individuals approach and solve mathematical tasks. As summarized below, eye-tracking research across various arithmetic domains—including simple arithmetic, complex multi-digit calculations, and multi-term arithmetic expressions—uncovers distinct cognitive processes, with gaze patterns providing a direct window into how attention is allocated to meet specific processing demands.

#### 3.2.1. Simple Arithmetic

Simple arithmetic facts generally refer to basic addition and multiplication problems involving single-digit operands (e.g., 6 + 2, 3 × 4), as well as their corresponding inverse problems from subtraction and division (e.g., 8 − 2, 12 ÷ 4). According to most cognitive models, arithmetic facts (mainly addition and multiplication) are stored in semantic memory as a result of overlearning, and can be accessed directly from there (for a review, Domahs & Delazer, 2005). However, research indicates considerable variability in how individuals solve these arithmetic facts across different operations (Campbell & Xue, 2001). Performance metrics such as RTs and accuracy rates are influenced not only by an individual’s proficiency but also by specific problem characteristics such as the magnitude of the operands and solutions, a phenomenon known as the *problem-size* effect (Zbrodoff & Logan, 2005). Assuming that eye movements, as measured by eye-tracking, can provide insight into the cognitive processes involved in mental arithmetic, Curtis et al. (2016) investigated how participants solve visually presented problems from one of the four basic operations verbally. Their goal was to establish whether gaze patterns could reveal the underlying solution strategies and the characteristics of the stored memory representations. Results indicated a distinct progression of fixations across operations. For addition, multiplication, and small subtraction problems, participants initially focused on the operator, gradually shifting their attention toward the right operand. In contrast, fixation time for large subtraction problems was more evenly distributed across all elements of the operation. During division, participants first focused on both the operator and the left operand, then exhibited a left-dominant fixation pattern. This tendency was more pronounced with larger problems. There was also some evidence that the operand order in addition and multiplication may influence fixation patterns. Overall, these results suggested that the problem characteristics such as problem size and operand order may influence visual attention distribution and that different cognitive processes are likely involved across the four basic operations. Fixation on the operator during addition, multiplication, and small subtraction problems may reflect memory retrieval. Conversely, distribution of attention to the relevant operand or operands in the case of division or large subtraction problems may indicate alternative strategies involving multiple steps and more effortful problem-solving processes. Evidence suggesting that eye-movement patters may reflect how arithmetic facts—namely, addition and multiplication—are stored in long-term memory, and whether operand order influences retrieval, has also been obtained by Zhou et al. (2012). They used a different eye-movement recording technique, namely an electrooculography. In their study, gaze patterns were specific to the operation and operand order, supporting the hypothesis that representations of addition and multiplication facts are dissociated in long-term memory and that the operand order in which these facts were learned may influence retrieval.

Similarly, Huebner and LeFevre (2018) used eye-tracking to investigate individual differences in solving simple subtraction problems. Specifically, their study examined whether distinct solution approaches were associated with systematic differences in eye-movement patterns for small (e.g., 5 − 3) and large (e.g., 14 − 6) problems. Participants were categorized as either *retrievers* (relying more on memory retrieval) or *procedure users* (relying more on transformation and counting) based on an analysis of ex-Gaussian parameters (mu and tau) from RT distributions, partly supplemented by self-report data. Analyses of total gaze duration and fixation counts revealed differential eye-movement patterns: retrievers focused primarily on the operator in small problems but evenly distributed their attention across problem components in large problems (for similar results in addition and multiplication, see Curtis et al., 2016). In contrast, procedure users evenly distributed their attention across problem components in small problems but focused significantly longer on the operands (particularly the right operand) in large problems. Thus, the gaze patterns of participants who used more procedural strategies differed from those of retrievers. In general, eye-movement patters were influenced by both the problem size and the preferred solution strategies, indicating that different approaches to solving a problem elicit distinctive patterns of attention allocation.

The “visual world paradigm” (Cooper, 1974) is an eye-tracking method in which a visual scene and its corresponding auditory description are presented simultaneously. In language research, this method has demonstrated that people instinctively look at the auditorily referenced objects, with their eye movements reflecting the rapid time course of comprehension (Altmann & Kamide, 2007; Tanenhaus et al., 1995). Hartmann et al. (2018) adapted this paradigm to study arithmetic problem solving by presenting a visual scene containing different numbers alongside an auditory arithmetic problem (e.g., 2 + 5, 7 − 5). They recorded the spontaneous fixation behavior while participants determined whether the problem’s result was among the presented numbers. On the visual display, numbers could be arranged either in ascending order or in descending order. Additionally, two of the numbers matched the operands of the auditorily presented arithmetic problem, one was an irrelevant number that served as “distractor” operand, and one number was either the correct or an incorrect solution of the arithmetic problem. Analysis of the mean fixation proportions revealed that eye movements closely mirrored participants’ ongoing cognitive processing—specifically, what they were currently considering mentally. Participants tended to fixate on the spoken numbers, anticipated potential operands, and eventually fixated on the computed solution (for similar results with a similar paradigm, see Hintz & Meyer, 2015). Additionally, the study revealed a significant association between problem-solving speed and eye-movement patterns: faster responses were linked to shorter latencies before fixating on relevant numbers and fewer revisits to the first operand during calculation. Eye-movement patterns were also influenced by the visual layout manipulations, showing that an ascending order of numbers improved performance in addition problems, while a descending order enhanced performance in subtraction problems. This indicated that the spatial organization of information can facilitate or hinder arithmetic processing, possibly by aligning with mental representations or simplifying search strategies. In summary, this study confirms once again that gaze patterns during arithmetic problem solving, as measured by eye-tracking, are not random but are tightly coupled with the underlying solution strategies.

#### 3.2.2. Complex Arithmetic

Complex, multi-digit calculations often extend beyond arithmetic fact retrieval, requiring the use of computational procedures and algorithms (Delazer, 2003). One such procedure is the *carry operation*, necessary when the sum of the digits in a given place value exceeds nine. For example, in the problem 47 + 28, the sum of the unit digits requires carrying a “one” over to the sum of the decade digits. Research shows that problems requiring carrying are more challenging than those that do not, as evidenced by longer response times (Geary & Widaman, 1987; Green et al., 2007; Widaman et al., 1989). Beyond such problem characteristics, studies demonstrate that arithmetic performance is also influenced by individual factors such as proficiency, age, and—crucially—the selection and execution of solution strategies. Participants of all ages employ diverse strategies, including counting, transformation, and retrieval, for problems of all complexity levels (Hinault & Lemaire, 2016; Lemaire, 2010). Variations in arithmetic performance based on problem type and participant characteristics can be explained by differences in cognitive demands, the strategies used, and how these are implemented across different problems (Hinault & Lemaire, 2016; Imbo et al., 2007). Eye-tracking methodology has been instrumental in elucidating these effects in complex mental calculation, moving beyond “pure” behavioral data.

For example, in a study by Moeller et al. (2011b), eye-tracking was used while participants performed a verification task involving multi-digit addition problems presented visually, some with carry and some without (e.g., 18 + 27 and 13 + 32). The results demonstrated that the need for a carry operation affects both early (encoding/estimation) and late (plausibility checking/calculation) processing stages. Notably, specific elements of the problem received increased attention during the processing of carry problems. More specifically, the unit digits of the addends (particularly the unit digit of the second addend) and the decade digit of the solution probe were linked to longer first fixation durations, a greater number of regressive saccades (i.e., leftward saccades to parts of the problem that had already been processed), and longer total reading times in carry problems. Further detailed analyses revealed that, in the initial stages, participants relied primarily on the exact calculation of the sum of the unit digits rather than approximate estimation to determine whether a carry operation was necessary. Based on these findings, the authors suggested that the difficulty associated with processing a carry problem is likely related to the increased cognitive load involved in accurately evaluating the unit digits and managing the additional step of carrying over to the next place value (for a similar eye-tracking study with children and adults, Moeller et al., 2011a).

Eye-tracking techniques were also employed to investigate age-related differences in strategy use during the solution of carry and non-carry problems. Green et al. (2007), for example, instructed both young and older adults on the use of the unit and hundred strategies. In three-digit addition problems (e.g., 519 + 461), the *unit strategy* involves adding the unit digits first, then the tens, and finally the hundreds. Conversely, the *hundred strategy* entails adding the hundreds first, then the tens, and finally the units. Participants completed the production tasks in two conditions: a choice condition (where they could select either strategy), and a no-choice condition (where they were required to apply a specified strategy) while their eye movements were recorded through eye-tracking. Behavioral analyses revealed group differences in accuracy and strategy selection, although RTs did not significantly differ. Older adults committed more errors and were less flexible in their strategy choices compared to young adults, aligning with findings of other studies on age-related effects in problem-solving strategies (Hinault & Lemaire, 2016; Lemaire & Arnaud, 2008; Taillan et al., 2015). Notably, the increased use of the unit strategy with a higher number of carries was greater in young people than in older adults. Typically, slower RTs were observed when people employed the unit strategy compared to when they employed the hundred strategy. Eye movements were affected by both carry effects and strategy use. Cumulative eye fixation durations were longer on carry than on non-carry problems, likely reflecting calculation and increased processing demands. Additionally, cumulative durations were longer on the hundred digits when employing the hundred strategy, and longer on the unit digits when using the unit strategy, suggesting that eye-movement patterns aligned with the strategy required to use or reported using. This effect was particularly more evident in young participants compared to older participants, indicating less differentiation between strategies in older adults. Results of this study suggested that carry effects may also stem from participants employing different sets of strategies, as problems with more carries were associated with increased use of the unit strategy. The use of the slower strategy, combined with additional processing demands, may likely contribute to poorer performance on carry problems compared to non-carry problems.

Inter-individual differences in strategy use are also evident in computation estimation (Ganor-Stern, 2015). In estimation tasks, individuals generate approximate answers rather than exact solutions—a crucial skill in daily life when time or attention is limited. Ganor-Stern (2015) identified three strategies during an estimation comparison task involving multi-digit multiplication problems (e.g., 37 × 54, is the estimated answer to the problem smaller or larger than the reference number 3900?): *approximate calculation* (rounding and multiplying), *sense of magnitude* (intuitive magnitude judgement), and *digits strategy* (comparing digit counts). The first two are more frequently used, with the approximate calculation strategy generally yielding higher accuracy and the sense of magnitude leading to quicker responses. College students typically report using more than one strategy simultaneously and demonstrate adaptive strategy use depending on problem characteristics, such as the proximity of the reference number to the correct answer (distance effect) and the relative size of the reference number compared to the actual answer (size effect; Ganor-Stern, 2015, 2016). Ganor-Stern and Weiss (2016) adopted eye-tracking methods to investigate how problem-solving strategies change with practice. Their findings revealed significant effects of distance, size, and practice. Specifically, as participants practiced, performance improved, as evidenced by shorter dwell time and faster responses. Dwell time was influenced by the relationship between the reference and the exact numbers: it was shorter when the reference number was far from the exact number (vs. close), and shorter when the reference number was smaller than the exact number (vs. larger). Notably, two problem-solving strategies were associated with distinct eye-movement patterns. The approximate calculation strategy was characterized by longer dwell time on the operands relative to the reference number, whereas the sense of magnitude strategy showed comparable dwell time on both the operands and the reference number. A detailed temporal analysis indicated that dwell time on the operands decreased over practice, while dwell time on the reference number increased, suggesting a shift in strategy from approximate calculation toward a more intuitive sense of magnitude with practice, in particular in the far condition. Overall, these findings demonstrated that eye movements are sensitive to problem characteristics, practice effects, and underlying strategy use, aligning with prior research by Green et al. (2007).

#### 3.2.3. Multi-Term Arithmetic Expressions

Gaining insight into how people process multi-term arithmetic expressions is important for elementary education, as well as for understanding the cognitive mechanisms at play in more advanced mathematics, such as algebra (Harrison et al., 2020; Rivera & Garrigan, 2016). Evaluating written arithmetic expressions involving multiple operators relies on learned procedural rules, but is also influenced, to some extent, by visual perception. Arithmetic processing requires mastering a set of specific procedural rules that determine the hierarchy and sequence of operations—such as addition, multiplication, and parentheses—in multi-term arithmetic expressions. For example, the *precedence* rule dictates that multiplication must be solved before addition in the expression 3 + 3 × 6. These rules are usually learned by rote at school and often appear idiosyncratic, as they do not always directly reflect the intrinsic meanings of the operations. Research suggests that applying these rules is partly guided by visual-perceptual/attentional factors. Landy et al. (2008) employed eye-tracking to empirically investigate a visually driven model of multi-term arithmetic computation, known as the *salience* model, originally proposed by Landy (2007). This model predicts that multiplication signs draw more visual attention than addition signs, consistent with the precedence rule, and that problems with narrowly spaced operands (e.g., 8 × 3) attract more attention than those with wider spacing (e.g., 8 × 3), as physical proximity facilitates the visual parsing and grouping of related elements. In general, their findings demonstrated that narrow spacing and multiplications are high-salience syntactic markers and have similar effects on attention and, consequently, on eye-movement patterns. However, the analysis of first fixations indicated that this bottom-up process is dynamically integrated with top-down parsing strategies. These strategies are deployed following an initial, rapid assessment of the overall structure of the expressions, revealing a complexity in the cognitive processes involved that the initial salience model could not account for.

Building on this work, Schneider et al. (2012) further investigated the nature of this syntactic parsing in multi-term arithmetic expressions using eye-tracking. Their main question was whether arithmetic syntax is processed sequentially, like language, or extracted in a rapid, parallel manner. In a series of experiments using multi-term arithmetic expressions with different syntactic trees (left-branching, right-branching, and center-embedded), the syntactic structure was indicated either explicitly (through brackets and/or parentheses, e.g., “n4 ± ((n1 ± n2) ± n3)”) or implicitly (via the precedence rule, e.g., “n4 + (n1 * n2 + n3)”). The results revealed a consistent initial fixation pattern across all expressions, reflecting a leftward bias. However, subsequent fixations rapidly adapted to each expression’s specific syntactic hierarchy, starting at the deepest nested level before proceeding through the remaining arithmetic sequence. Critically, eye-movement patterns were not affected by whether the syntactic structure was conveyed by explicit visual cues (brackets and parentheses) or by implicit rules (multiplication first). This was consistent with the view that, unlike language where a sentence has to be scanned progressively while the syntax is extrapolated sequentially, syntax in arithmetic can be extracted very quickly and in parallel.

Taken together, eye-tracking studies provide a valuable window into how people solve arithmetic problems, across simple facts, complex multi-digit calculations, and multi-term expressions. Across these domains, gaze patterns reveal whether participants rely on memory retrieval, procedures and rules, or strategic approaches, and how problem features shape processing dynamics. Eye-tracking thereby offers a reliable, complementary method for investigating the procedures and solution strategies used during arithmetic problem solving, addressing the limitations of self-reports, which can suffer from participants’ insufficient insight into the strategies they actually employ (Green et al., 2007; Smith-Chant & LeFevre, 2003).

## 4. Discussion and Perspectives

The body of eye-tracking research reviewed here underscores the substantial value of this methodology in investigating the cognitive underpinnings of adult mental arithmetic. By moving beyond traditional behavioral measures such as RTs, accuracy, and verbal reports, eye-tracking provides a spatially and temporally precise record of the attentional and strategic processes underlying mental calculation. The evidence converges on two primary, albeit interconnected, conclusions: first, that spatial attention is dynamically and flexibly engaged during arithmetic processing; and second, that the procedures, rules, and strategies individuals use are directly reflected in the allocation of visual attention.

The findings on spatial-arithmetic associations align with a view of a complex, context-dependent cognitive framework. Converging with the insights from behavioral studies (e.g., Liu et al., 2017; Masson & Pesenti, 2014, 2016; Pinhas & Fischer, 2008), eye-tracking studies support a dynamic and flexible model where spatial-arithmetic mappings are bi-dimensional (Salvaggio et al., 2022) and are modulated by task demands (Hartmann, 2022; Klein et al., 2014), problem structure (Zhu et al., 2019), and problem difficulty (Salvaggio et al., 2022). However, their precise time course still remains an open question. Some eye-tracking studies report spatial attention shifts immediately after encoding the operator (Hartmann et al., 2015), whereas others locate them during the computation of the answer (Masson et al., 2018). Thus, while spatial attention shifts during mental arithmetic are clearly not merely epiphenomenal, their exact functional role remains unclear. Many experiments employ different paradigms, each with differing objectives and control conditions. Some paradigms explicitly manipulate spatial variables (e.g., number line orientation), while others do not. This heterogeneity contributes to inconsistent results and hampers the development of a unified explanation about the functional significance of spatial attention shifts during mental calculation.

Simultaneously, eye-tracking research on procedures, rules, and solution strategies has detailed how visual attention is allocated across problem components. In general, fixations are longer and more frequent on elements relevant to the solution (Hartmann et al., 2018). Fixation patterns, dwell times, and saccades differentiate memory retrieval from computation (Curtis et al., 2016; Huebner & LeFevre, 2018), carry from non-carry problems (Green et al., 2007; Moeller et al., 2011b), and strategy selection (Green et al., 2007; Huebner & LeFevre, 2018). These effects interact with problem characteristics such as operation type (Curtis et al., 2016), problem size (Curtis et al., 2016; Huebner & LeFevre, 2018), and syntactic structure (Landy et al., 2008; Schneider et al., 2012). Taken together, these findings confirm that eye-tracking measures are tightly coupled with strategy execution and provide objective insights that complement—and often surpass—the reliability of verbal self-reports (Huebner & LeFevre, 2018). As also shown by Susac et al. (2014) (see also Chesney et al., 2013) in a study on algebraic equation solving, eye-tracking measures can reveal participants’ problem-solving efficiency and solution approaches, which are often difficult to capture through verbal report alone, especially for those participants with less self-insight. Furthermore, eye-tracking measures demonstrate how strategy use evolves with practice (Ganor-Stern & Weiss, 2016) and what factors influence parsing of arithmetic syntax (Schneider et al., 2012; Landy et al., 2008).

Although the results presented in this review are encouraging, the reliability of some observed effects should not be overinterpreted. Methodological factors—such as drift correction procedures, data processing choices, stimulus design, and synchronization methods—can significantly influence outcomes and potentially bias the observed eye-movement patterns. Therefore, further research with rigorous and standardized methods is necessary to confirm and strengthen these findings. Moreover, it should be acknowledged that very similar eye movement patterns can correspond to very different underlying cognitive processes. For example, a prolonged fixation on a multiplication sign could reflect its high perceptual salience, arithmetic fact retrieval from memory, or increased computational effort for a difficult operation. The eye-tracking data alone do not allow for such a differentiation. Converging evidence from multiple methodologies is thus essential for a deeper understanding of the cognitive architecture underlying arithmetic processing.

**Figure 1:**
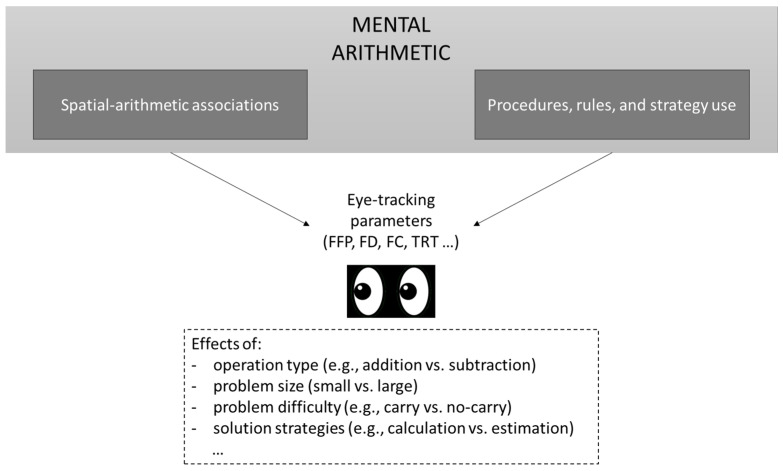
Schematization of the aspects in arithmetic processing that may be investigated through eye-tracking. Legend. FFP = first fixation position, FD = fixation duration, FC = fixation count, TRT = total reading times.

In summary, eye-tracking provides a rich source of data on different aspects of arithmetic processing (see Figure 1). A range of eye-tracking measures enable a fine-grained investigation of mental arithmetic (for a discussion, see Mock et al., 2016) and are sensitive to manipulations of operation type, problem size, problem difficulty, or solution approaches. Notably, eye-tracking can also be used to investigate individual differences in solution approaches and arithmetic proficiency (Huebner & LeFevre, 2018; Susac et al., 2014), as well as to control for participants’ compliance with instructions (Hartmann, 2022). Research on mental arithmetic in children has strongly demonstrated the value of eye-tracking for investigating individual differences in mathematics. For instance, studies have revealed significant correlations between fixation patterns and task performance (Hunt et al., 2015; Porras et al., 2024), as well as with levels of math anxiety (Hunt et al., 2015). Specifically, a higher number of fixations and longer fixation durations on operands are associated with lower arithmetic performance (Porras et al., 2024) and higher math anxiety (Hunt et al., 2015). Furthermore, studies have identified distinctive fixation patterns in children with dyscalculia—a condition characterized by deficits in number processing—that differ markedly from their typically developing peers (Van Viersen et al., 2013). The potential of eye-tracking lies in its ability to detect subtle differences in performance both within individuals and across different populations. This approach holds substantial promise for advancing our understanding of arithmetic processing across the lifespan, from typically developing children to healthy adults and clinical populations. Crucially, by identifying specific impaired processing stages, eye-tracking can directly inform the development of targeted interventions to improve mathematical skills. As shown by the studies reviewed here, eye-tracking is exceptionally well-suited for investigating individual differences in arithmetic ability, strategy efficiency, and cognitive load. Future work should explore how gaze patterns differ in individuals with arithmetic difficulties (e.g., brain-damaged patients with acalculia). This could lead to the development of eye-tracking-based diagnostic tools to identify specific deficits (e.g., ineffective carry processing) and to evaluate the effectiveness of different cognitive interventions by tracking changes in visual attention and arithmetic performance.

In conclusion, the research reviewed here shows that eye-tracking goes beyond traditional behavioral measures by providing a direct window into the cognitive processes of arithmetic, rather than merely its behavioral products. The converging evidence confirms that spatial attention and solution strategies are fundamental, measurable components of calculation. As such, eye-tracking has moved beyond a complementary technique to establish itself as a useful tool for building a more nuanced understanding of mathematical cognition and its impairments.

## Data Availability

No new data were created or analyzed in this study. Data sharing is not applicable to this article.
